# Air and Water Environmental DNA Enable Detection of a Large Terrestrial Bird: A Case Study of the Southern Cassowary (*Casuarius casuarius*)

**DOI:** 10.1002/ece3.74578

**Published:** 2026-10-23

**Authors:** Peta Hill, Simon Booth, Daniel Guymer, Ariella Matthews, Dinouk Perera, Matt Brien, Elise Furlan

**Affiliations:** ^1^ Institute for Applied Ecology University of Canberra Bruce Australian Capital Territory Australia; ^2^ Queensland Government Department of the Environment, Tourism, Science and Innovation Brisbane Queensland Australia; ^3^ Queensland Government Department of Primary Industries Brisbane Queensland Australia; ^4^ NatureDNA Port Macquarie New South Wales Australia

**Keywords:** conservation, eDNA, frugivore, monitoring, non‐invasive, rain‐forest

## Abstract

The southern cassowary, *Casuarius casuarius* , is a large‐bodied terrestrial frugivore that plays a critical role in tropical rainforest ecosystems but can be challenging to locate and survey using traditional methods. Across much of its range, populations are threatened by habitat loss, fragmentation and other anthropogenic pressures, and key aspects of distribution and movement remain poorly understood. Environmental DNA (eDNA) offers a non‐invasive approach to monitoring elusive species, yet applications for large, mobile terrestrial vertebrates—particularly using air as a sampling medium—remain limited. Here, we develop and validate a highly sensitive, species‐specific TaqMan‐based eDNA assay to detect the southern cassowary and evaluate its performance using both air and water samples collected in captivity and in the field at sites of known presence. Cassowaries represent a biologically challenging test case for eDNA detection due to their mobility, low local densities and terrestrial ecology. Despite this, we demonstrate that eDNA from both air and water sources provides a practical, non‐invasive tool to monitor this keystone rainforest species across landscapes where traditional surveys are logistically constrained. Importantly, this approach provides a scalable method for rapidly assessing cassowary presence, supporting landscape‐scale conservation planning and management. Our results highlight the potential of airborne eDNA for detecting large terrestrial vertebrates, with broader implications for biodiversity monitoring.

## Graphical abstract

The southern cassowary is a large‐bodied, endangered rainforest frugivore that plays a critical ecological role in tropical forest dynamics. We developed and validated a highly sensitive, species‐specific eDNA assay for the southern cassowary. We evaluate the assay's performance using both air and water samples collected in captivity and in field sites of known occupancy in Australia. Despite cassowaries representing a biologically challenging target for eDNA detection, we demonstrate that cassowary DNA can be reliably detected from both environmental sample types under real‐world conditions.

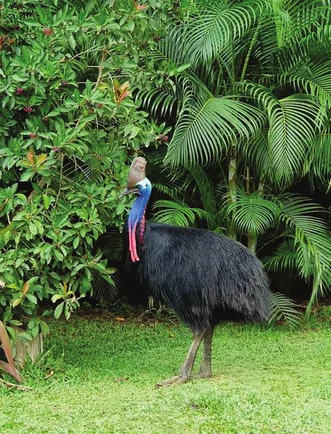

## 1 Introduction

The southern cassowary, *Casuarius casuarius* , is Australia's heaviest flightless bird and the country's only cassowary species (Figure 1). It occupies the tropical rainforests of Queensland's north and is divided broadly into three disjunct populations: one in the wet tropics and two in Cape York (Bradford and Westcott 2010; Westcott et al. 2014). Southern cassowaries are frugivores and play a critically important role as seed dispersers within tropical rainforests (Mahoney et al. 2018; Albert et al. 2020). As Australia's only large‐bodied frugivore, they are capable of dispersing large quantities of seeded fruits over significant distances, contributing to rainforest composition and structure (Bradford et al. 2008; Campbell et al. 2023). The southern cassowary also occurs in Indonesia and Papua New Guinea where it shares parts of its range with the northern cassowary ( *C. unappendiculatus* ) and the dwarf cassowary ( *C. bennetti* ) (Dominy et al. 2018). Southern cassowaries can grow to 1.8 m tall and weigh up to 80 kg (Campbell et al. 2014), yet despite their large structure and distinctive morphology (brightly coloured neck and prominent casque on the head), the species is often challenging to detect and survey due to its cryptic behaviour and the dense, structurally complex habitats it occupies (McLean et al. 2017).

**FIGURE 1:**
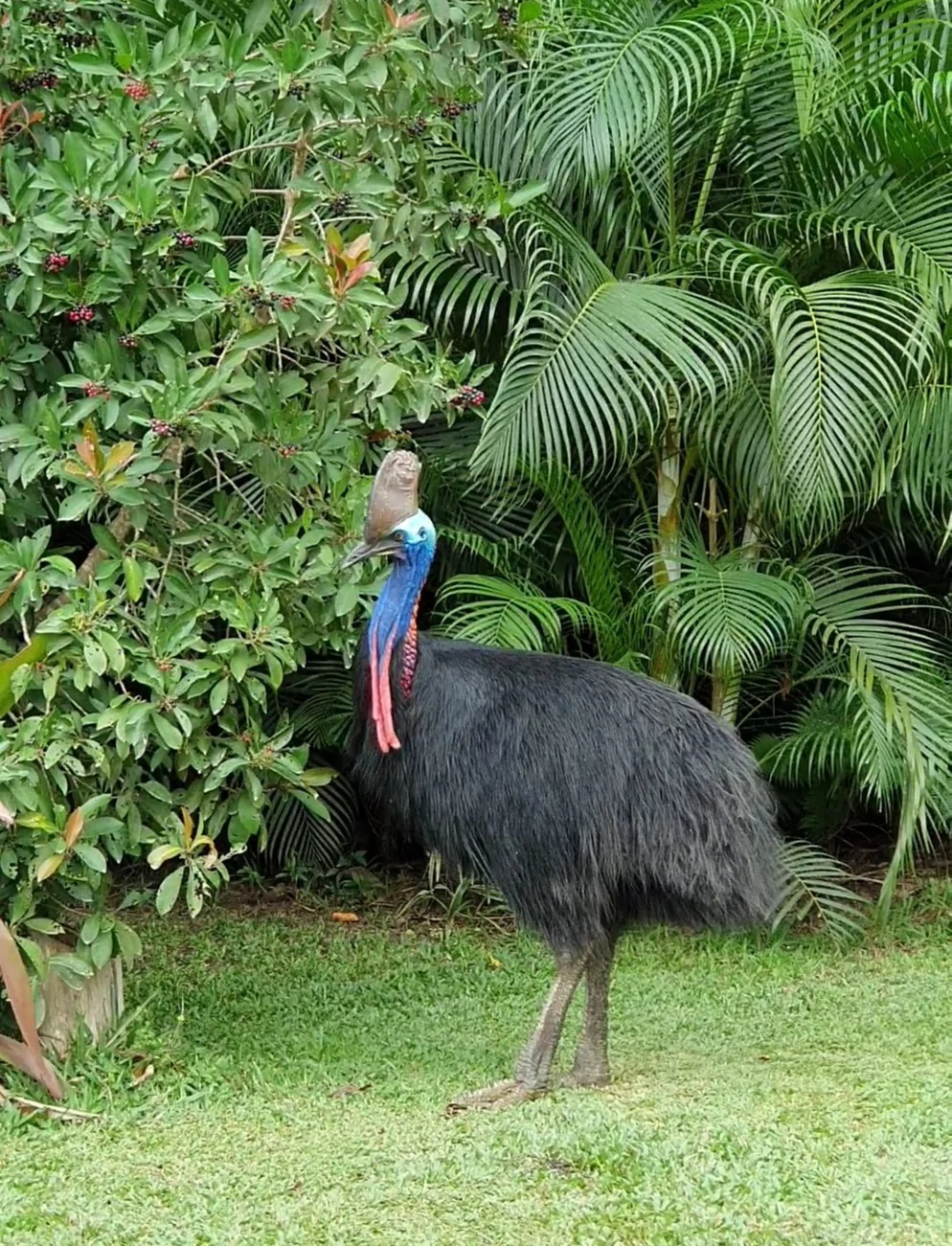
A large mature southern cassowary, *Casuarius casuarius*, from the wet tropics region in Queensland, Australia.

In Australia, the southern cassowary is listed as endangered and faces multiple threats, including climate‐driven habitat loss, habitat fragmentation and road induced mortality (Dwyer et al. 2016; Dennis and the Cassowary Recovery Team 2023). Vehicle strikes represent a particularly significant source of mortality, accounting for 39 of 53 recorded deaths in the wet tropics population between 2023 and 2025 (Department of the Environment, Tourism, Science and Innovation [DETSI], unpbl. data). Historic clearing and degradation of rainforest for sugar cane plantations, cattle grazing and urbanisation across the Australian wet tropics bioregion, which covers an area of 8944 km^2^ (Stork et al. 2008; Campbell et al. 2023), have resulted in declines in forest biodiversity and significant reductions in lowland rainforest habitats. Although the species typically inhabits these lowland rainforest areas, they also utilise a wide range of resources that may be available, including melaleuca, palm and mangrove swamps, beaches, woodlands, eucalypt forests and suburban gardens (Dennis and the Cassowary Recovery Team 2023).

Southern cassowaries are naturally mobile across the landscape to access the variety of resources they require, with reported home range utilisation < 1 km^2^ for males, and two to three times larger for females (Campbell et al. 2014), although this varies according to habitat quality, structure, resource availability and population density. Despite ongoing threats, the wet tropics population appears to be stable. Less, however, is known about the status of the remote Cape York population, including the severity of threats, the extent of their range and possible connectivity between populations (Dennis and the Cassowary Recovery Team 2023). Improved methods for detecting the species, particularly in remote and low‐density contexts, are therefore required.

Environmental DNA (eDNA) surveys rely on detecting a species from trace amounts of DNA shed into their environment through sloughed cells, faeces or urine and provide a valuable technique for detecting rare, elusive and invasive species in both aquatic and terrestrial environments (Ficetola et al. 2008; Bylemans et al. 2017; Eiler et al. 2018; Rojahn et al. 2018; Beng and Corlett 2020; Villacorta‐Rath et al. 2020). While eDNA applications in avian monitoring lag behind those for other taxa (Ke et al. 2025), they have proven effective for detecting birds that are elusive or occur at low densities (Day et al. 2019; Neice and McRae 2021; Guan et al. 2023; Young et al. 2025).

The southern cassowary is known to spend time in and around water including upland streams and forages in estuarine areas. As such, eDNA collection from water samples (McElroy et al. 2020) may provide a valuable means of detecting species presence. Recently, eDNA collection from air has emerged as a promising approach for biodiversity monitoring (Lynggaard et al. 2022; Johnson et al. 2023; Ke et al. 2025) and species‐specific detection (Garrett et al. 2023). Airborne eDNA may be particularly well‐suited to large‐bodied birds such as cassowaries as they can release substantial amounts of skin cells when they preen or fluff their feathers, resulting in airborne DNA available for capture. In this study, we develop and validate a species‐specific TaqMan‐based eDNA assay to detect the southern cassowary in Australia. We assess assay specificity to the target species and sensitivity to detect down to very low concentrations of DNA, typical of eDNA samples. We evaluate the performance of the assay using airborne eDNA alongside concurrent water sampling taken in captivity and in the field from sites of known cassowary presence. By enabling detection across both aquatic and terrestrial environments, this approach has the potential to improve spatial coverage of surveys in heterogeneous rainforest landscapes where traditional monitoring methods are often constrained.

## 2 Materials and Methods

### 2.1 Study Species and Co‐Occurring Species

The southern cassowary is a large flightless bird. The species has a limited distribution throughout the tropical rainforests of far north Queensland, Australia in addition to being widespread throughout Indonesia and Papua New Guinea. The closest relative to the southern cassowary in Australia is the emu ( *Dromaius novaehollandiae* ; Mitchell et al. 2014) and there is almost no range overlap between these species (Sales 2009). In Papua New Guinea and Indonesia, the southern cassowary range has limited overlap with that of the northern cassowary ( *C. unappendiculatus* ) and the dwarf cassowary ( *C. bennetti* ) (Dominy et al. 2018).

### 2.2 Assay Development and Validation

#### 2.2.1 Sequence Database Generation

All readily available mitochondrial DNA (mtDNA) sequence records for the Cytochrome b gene of the southern cassowary (*n* = 18) and the closely related emu (*n* = 3), were collated from online databases (Benson et al. 2009) and imported into Geneious Prime 2022.2.2 (https://www.geneious.com). The southern cassowary sequences included samples originating from both Australia and Papua New Guinea (Table S1). Sequences were aligned using the CLUSTAL OMEGA algorithm. Sequencing primers were designed to sequence a ~832 bp region of the mitochondrial Cytochrome b gene in the southern cassowary. Primers C.cas_CytB_1F (5′‐GCTCCCTAATTGATCTCCCTTC‐3′) and C.cas_CytB_1R (5′‐GCAAGTACGCCTCCTAGTTTAT‐3′) were designed using the Primer 3 function in Geneious Prime 2022.2.2 (https://www.geneious.com). Annealing temperature (Tm), GC content and secondary structure of primers and probe were checked using ThermoFisher and Integrated DNA technologies (IDT) online tools (Integrated DNA Technologies, n.d.; ThermoFisher Scientific, n.d.).

An additional 56 Australian southern cassowary tissue samples were obtained to account for intraspecific variation in sequences during the assay design. These samples were collected opportunistically, predominately from vehicle strikes or deceased individuals located throughout the wet tropics region of Queensland (Figure S1). Samples were collected between July 2018 and July 2022. An additional two emu tissue samples were obtained from the Australian National Wildlife Collection (ANWC) to monitor for non‐specific amplification in this closely related species. DNA was extracted from these tissues by Diversity Arrays Technologies (DArT Pty Ltd., Canberra, Australia) using a NucleoMag 96 Tissue Kit (Macherey‐Nagel, Düren, Germany) coupled with NucleoMag SEP (Ref. 744,900) to allow automated separation of high‐quality DNA on a Freedom Evo robotic liquid handler (TECAN, Männedorf, Switzerland). Tissue was first incubated for 4 h to overnight with proteinase K, adjusted in concentration depending on the sample.

Extracted DNA was amplified using the newly generated primers in a PCR mastermix consisting of 10 μL × MyTaq Red Mix (Bioline), 0.4 μL (10 μM) forward primer, 0.4 μL (10 μM) reverse primer, 2 μL DNA and made up to 20 μL with UltraPure H2O (Invitrogen). Three no template controls were run on each plate. Thermocycling was conducted on a ViiA7 real‐time PCR machine (Applied Biosystems) at 95°C for 3 min followed by 40 cycles of 95°C for 30 s, 55°C for 30 s and 72°C for 45 s followed by a final extension at 72°C for 5 min. All PCR products were purified using the MinElute PCR Purification Kit (Qiagen Pty Ltd., Melbourne, Vic.) and sequenced on an AB 3730xl DNA Analyser (at the Biomolecular Resource Facility, The John Curtin School of Medical Research, Australian National University, Canberra, ACT, Australia). Sequences were checked and edited manually in Geneious Prime 2022.2.2 (https://geneious.com) to trim low‐quality bases from the 5′ and 3′ ends, align forward and reverse reads to generate consensus sequences, and resolve any discrepancies by inspecting the chromatogram traces, particularly in homopolymer regions where automated base calling can be unreliable. Sequences were then aligned with all previously downloaded sequences.

#### 2.2.2 Assay Design

The C.cas_Cytb assay was developed as a TaqMan assay which comprises forward and reverse primers and a FAM‐labelled minor groove binder (MGB) hydrolysis probe. The assay was designed to target a 116 bp region of the cytochrome b gene that incorporated genetic differences between the cassowary and the emu (Table 1). The assay was designed by the Primer 3 function in Geneious, ensuring that the primers and probe sequences were identical to all known southern cassowary sequences while maximising sequence variation with emu. Annealing temperature (Tm), GC content and secondary structure of primers and probe were checked using ThermoFisher and Integrated DNA technologies (IDT) online tools (Integrated DNA Technologies, n.d.; ThermoFisher Scientific, n.d.). The primers were then tested for specificity and cross‐amplification to non‐target species *in silico* using Primer‐BLAST (Ye et al. 2012), and the predicted probability of cross‐amplification for all identified non‐target species was assessed using eDNAssay (Kronenberger et al. 2022).

**TABLE 1:**
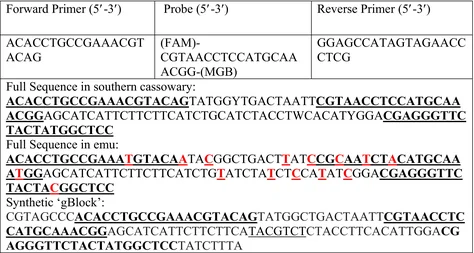
Details of primers and hydrolysis probe for the C.cas_CytB TaqMan assay designed to amplify a 116 bp region of the Cytochrome b mitochondrial gene for the southern cassowary.

*Note:* The full sequence of the region of interest is also shown for southern cassowary and emu (with sequence mismatches to southern cassowary in bold red underline) and for the synthetic ‘gBlock’. Primer and probe locations are shown in bold underline with gBlock inversion section underlined.

#### 2.2.3 Assay Specificity and Sensitivity

The TaqMan C.cas_CytB assay was tested *in silico* by comparing the primer and probe sequences to all available southern cassowary and emu sequences and for amplification using genomic DNA from four southern cassowary and two emu individuals. Real‐time PCRs were performed in duplicate 20 μL reactions containing 1 μL of 20X C.cas_CytB TaqMan assay, 10 μL of Taqpath ProAmp master mix, 2 μL of template and 7 μL of ultra‐pure water. PCR cycling was conducted on an Applied biosystems QuantStudio 7 Pro with thermal cycling conditions set at 50°C (2 min), 95°C (10 min), followed by 50 cycles of 95°C (15 s) and 58°C (1 min). Samples were classified as positive if exponential amplification occurred within the first 40 cycles in at least one technical replicate.

The sensitivity of the assay was determined using a synthetic oligonucleotide (gBlock; https://sg.idtdna.com/). This double stranded synthetic Cytochrome b gene fragment was designed to match the southern cassowary Cytochrome b sequence with 8 bp overhang at either end and a 7 bp inversion to enable detection of any carry over contamination (Table 1). A dilution series of this oligonucleotide was prepared to generate concentrations between 0.1 and 1,000,000 copies per microlitre. Ten replicate PCRs were performed for each concentration and amplified with the same chemistry and conditions as the specificity testing. Cq values were then used as per Klymus et al. (2020) to calculate the limit of detection (LOD) for this assay.

### 2.3 eDNA Validation

Two eDNA validation studies were performed to confirm detection of southern cassowary DNA from air and water samples. eDNA samples were collected from a semi‐natural environment within fenced enclosures containing *C. casuarius* . Air sampling occurred at Garners Beach Cassowary Rehabilitation Facility in Garners Beach, Queensland, Australia and water sampling occurred at Hartley's Crocodile Adventures in Cairns, Queensland Australia (see Supporting Information S1 for further details).

#### 2.3.1 Airborne eDNA Sampling

Air samples were taken using custom air samplers designed and built at the University of Canberra. The custom air samplers capture airborne particulates on an F5‐rated fibreglass filter media (CC500; Vilair‐AAF), a medium‐efficiency filter designed to capture fine airborne particulate matter. Air was passed through the filter via a battery–operated suction fan controlled by a timer. The filters were collected at the end of each sampling period, rolled tightly, ensuring the collection surface faced inwards, and placed into a 5 mL sterile tube containing enough ethanol to saturate the filter (approximately 2 mL).

Within the *C. casuarius* enclosure at Garners Beach, three air sampling units were run for between 60 and 90 min (see Supporting Information S1; Figure S2 for further details).

#### 2.3.2 Water eDNA Sampling

Water samples were taken using custom kits comprising 5 μM polyethersulfone filters, one 50 mL syringe, 300 μL of DNA/RNA shield preservative (www.integratedsci.com.au) pre‐loaded in a 1 mL syringe, and gloves in a resealable pouch. Surface water was sampled using a bucket sterilised with 1% commercial bleach that was transported to the site in a new plastic garbage bag. Water was passed through each sample filter using repeat 50 mL aliquots until 1000 mL had been sampled or until the filter clogged. Following filtration, 50 mL of air was passed through the filter to remove residual water before the preservative was applied to the filter. The preservative syringe was left attached to the filter, the filter was capped and placed back in the resealable pouch. Equipment control/sample volume, date and trial details were recorded on the outside of the pouch.

Five water samples were taken from roughly the same location in a small concrete drinking water pond (approx. 1 m diameter) in the *C. casuarius* enclosure at Hartley's. One equipment control was also sampled, consisting of 750 mL of tap water from the food preparation tank containing water sourced upstream of Hartley's.

### 2.4 Field Study

Air and water sampling was undertaken in the Mission Beach region of Queensland, Australia, where cassowaries are regularly reported. Cameras were deployed at three field sites for up to 10 days to confirm southern cassowary presence before and/or during the field sampling (Table S2; Figure S3). Air samplers were positioned along paths regularly used by cassowaries, with three or four samplers deployed per site and spaced up to 43 m apart. Water eDNA samples were collected from nearby permanent, transient or flowing waterbodies located within 65 m of the air sampling sites. Additional water‐only samples were collected from four nearby sites where southern cassowaries had been recently observed. Further details of site locations, sampling schedules and site layouts are provided in the Supporting Information S1.

#### 2.4.1 Airborne eDNA Sampling

Air sampling took place on three sites, designated as sites T, A and B (see Supporting Information S1; Figure S3 for site details). Air samples were collected for either 1 day or three consecutive days depending on the site, with air sampled for 5 min every hour for 8 h a day. The filters were collected at the end of the 8 h. Multiple air sampling units were deployed at each site. Each unit was cable‐tied to a star picket approximately 2 m off the ground and covered partially with a small bucket for protection against the rain.

Positive and negative controls were collected using the same sampling scheme for three consecutive days. We again collected air samples from the Garners Beach Cassowary Rehabilitation Facility to use as our positive control using an enclosure that housed two to three birds during the study (Figure 2). A negative control air sampler was set up at the Cairns DETSI base located in the central business district of Cairns, Queensland where southern cassowaries are rare (Westcott et al. 2014). A single air sampling unit was deployed at both the positive and negative control sites.

**FIGURE 2:**
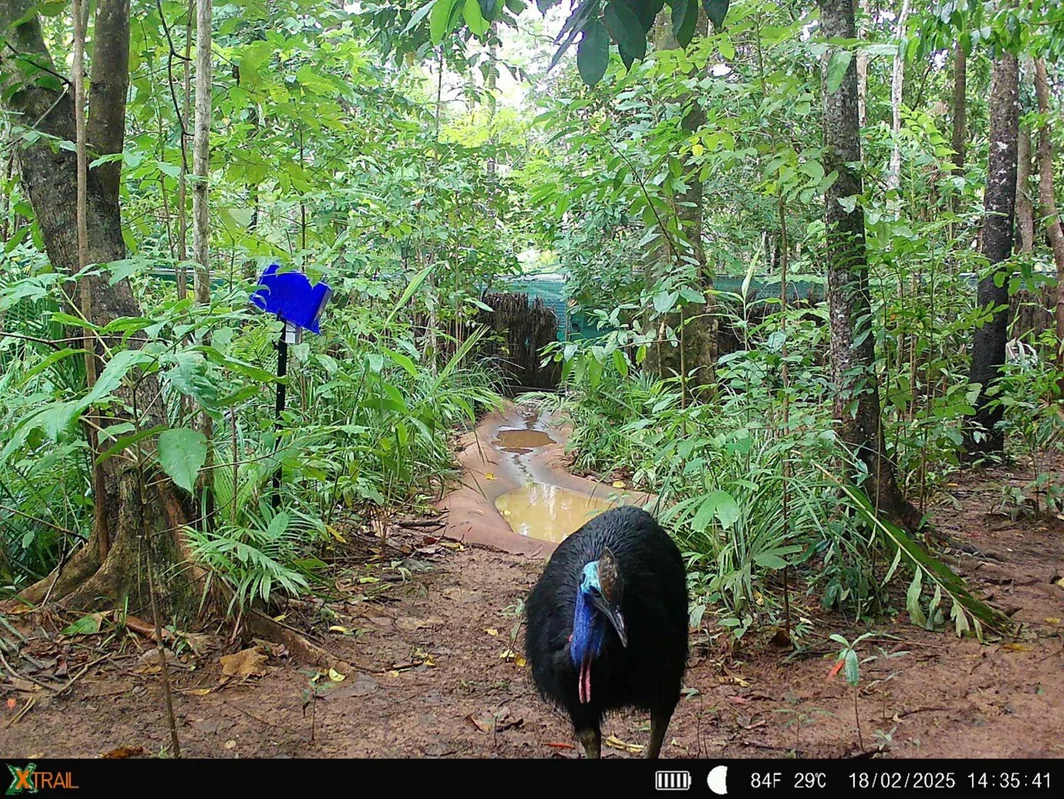
Trail camera photo of an air sampler mounted on a star picket in a rehabilitation enclosure at the Garners Beach Cassowary Rehabilitation Facility. Photo supplied by DETSI.

#### 2.4.2 Water eDNA Sampling

Water samples were taken at two of the air sampling sites in addition to four sites around Mission Beach where cassowaries are regularly reported (see Supporting Information S1; Table S2; Figure S3). Each water sample was filtered as described above using three separate filters used on each sampling occasion, with each filter representing an independent subsample that was extracted and analysed separately.

A positive control was taken from the Garners Beach Cassowary Rehabilitation Facility and a negative equipment control was taken at each site by pouring 750–1000 mL of bottled water into the sterile bucket and filtering as described.

### 2.5 eDNA Extraction, Amplification and Sequencing

All air and water samples were transferred to the University of Canberra, stored at −20°C and extracted within 2 months of collection. eDNA extractions were performed at the National eDNA Reference Centre's (https://www.ecodna.org.au/nrc) trace DNA facility in a biological safety cabinet. DNA was extracted from air and water samples using the Qiagen DNeasy blood and tissue kit following the manufacturer's directions (https://www.qiagen.com/au) with modifications to accommodate the sample types (see Supporting Information S1).

Extractions were performed in batches of no more than 11 samples at a time with an extraction control included with each batch. All eDNA samples, equipment controls and extraction controls were tested neat using the C.cas_Cytb TaqMan assay with the conditions outlined above (under *Assay specificity*) on the Applied Biosystems QuantStudio 7 Pro. Samples were tested in triplicate, and three no template controls and three positive controls were included on each plate. Positive amplifications were Sanger sequenced on an AB 3730xl DNA Analyser (at the Biomolecular Resource Facility, The John Curtin School of Medical Research, Australian National University, Canberra, ACT, Australia) to confirm amplification of the target species.

## 3 Results

### 3.1 Assay Development and Validation

#### 3.1.1 Sequence Database Generation

Primers designed to amplify an ~832 bp fragment of the mitochondrial cytochrome b gene had a GC content of 45%–50% and maximum Tm differed by only 0.05°C (57.99°C and 57.94°C for the forward and reverse primer respectively). All dimers were within the tolerable range (ΔG ≥ −4.0 kcal). All 56 newly sequenced cassowary samples shared an identical sequence, representing a previously unreported haplotype (GenBank Accession number PZ791489). As full‐length sequences were unavailable for many published cassowary samples, we compared an internal ~307 bp region of cytochrome *b* against a larger set of publicly available sequences (Benson et al. 2009), including individuals of the *beccarii*, *sclaterii* and *violicollis* subspecies (*n* = 20) and emu (*n* = 3). Within this region mean pairwise variation among cassowary sequences was 0.5% (range 0–6 nucleotide differences) and mean pairwise variation between cassowary and emu was 12.3% (range: 36–41 nucleotide differences).

#### 3.1.2 Assay Design

Primers and probe were designed with a perfect sequence match for all southern cassowary individuals while incorporating a total of seven mismatches to emu: two, one and four mismatches in the forward primer, reverse primer and probe respectively (Table 1). GC composition ranged between 55% and 57.1% and max Tm differed by < 1.3°C (60.60°C and 59.39°C for the forward and reverse primer respectively). All dimers were within the tolerable range (ΔG ≥ −3.4 kcal).

#### 3.1.3 Assay Specificity and Sensitivity

Primer‐BLAST identified possible cross‐amplification with three non‐target species co‐occurring in Australia, as well as possible amplification with a further eight non‐target species occurring within the cassowary's broader distribution in Papua New Guinea and Indonesia (between 4 and 7 mismatches across the primer‐probe regions, see Table S3). In silico cross‐amplification testing using eDNAssay (Kronenberger et al. 2022) found that all co‐occurring Australian taxa, including emu ( *Dromaius novaehollandiae* , AP = 0.231), fell well below the default 0.55 threshold and are not predicted to cross‐amplify with the cassowary‐specific assay. Across the assay's broader distributional range, one non‐target taxon, *Chalcopsitta scintillata* (dusky lorikeet, New Guinea), returned an assignment probability close to threshold (AP = 0.546).

In vitro testing of the assay amplified in all southern cassowary samples and failed to amplify in non‐target emu samples, as expected. The C.cas_CytB assay was found to be extremely sensitive and able to detect DNA concentrations for the target species as low as two copies per reaction (Tables S4 and S5). This assay can reliably detect 200 copies per reaction with a 95% detection (*R* ^2^: 0.998, efficiency: 91.3%, y‐intercept: 41.3 and slope: −3.67). The effective LOD for multiple PCR replicates for this assay was calculated following the R code from Klymus et al. (2020) (https://github.com/cmerkes/qPCR_LOD_Calc) and is reported in Table S5.

### 3.2 eDNA Validation

Cassowary DNA was detected in one out of three air samples taken from the Garners Beach Rehabilitation Facility and in all five water samples taken from Hartley's Crocodile Adventures (Tables S6 and S7). All control samples performed as expected.

### 3.3 Field Study

#### 3.3.1 Airborne eDNA Sampling

Cameras confirmed the presence of southern cassowaries at all three air sampling field sites. Cassowaries were recorded on camera at the site and date of each sampling event, with the exception of Site T on Day 2. Southern cassowary DNA was detected in air samples from two field sites: Day 3 at Site T and Day 1 (the only day of sampling) at Site A (Table 2). Southern cassowary DNA was detected on all three sampling days at the positive control site, and Sanger sequencing confirmed amplification of southern cassowary DNA in all positive amplifications tested. No southern cassowary DNA was detected in the air samples from Site B (1 day of sampling) or at the negative control site (3 days sampling).

**TABLE 2:** qPCR amplification success of air samples tested using the C.cas_CytB assay for the detection of southern cassowary at three sites around Mission Beach, Queensland, Australia.

Site code	Day 1				Day 2				Day 3			
	Amp. success (Cq mean)				Amp. success (Cq mean)				Amp. success (Cq mean)			
	Unit 1	Unit 2	Unit3	Unit4	Unit1	Unit 2	Unit3	Unit4	Unit1	Unit 2	Unit3	Unit4
Site T*	0/3	0/3	0/3	0/3	0/3	0/3	0/3	0/3	1/3 (38.4)	0/3	0/3	0/3
Site A	0/3	3/3 (37.0)	0/3	0/3	—	—	—	—	—	—	—	—
Site B*	—	—	—	—	—	—	—	—	0/3	0/3	0/3	—
Neg. control	0/3				0/3				0/3			
Pos. control*	3/3 (37.0)				3/3 (30.6)				3/3 (36.3)			

*Note:* ‘Unit’ refers to an individual air sampler. Where amplification occurred, the number of qPCR replicates are included and mean Cq is provided in parentheses. ‘—’ indicates no sampler was deployed. * indicates sites where water was also sampled. See Supporting Information S1 for site descriptions.

#### 3.3.2 Water eDNA Sampling

Southern cassowary DNA was detected in water samples from five out of six field sites and at the positive control site (Table 3). Sanger sequencing confirmed southern cassowary DNA in all positive amplifications. Southern cassowary DNA was not detected in any of the negative control samples.

**TABLE 3:** qPCR amplification success of water samples tested using the C.cas_CytB assay for the detection of southern cassowary at seven sites around Mission Beach, Queensland, Australia.

Site	Waterbody type	Subsample 1		Subsample 2		Subsample 3		Equipment control	
		Vol (mL)	Amp. success (Cq mean)	Vol (mL)	Amp. success (Cq mean)	Vol (mL)	Amp. success (Cq mean)	Vol (mL)	Amp. success (Cq mean)
T*	Permanent pool	400	3/3 (38.7)	350	0/3	300	1/3 (38.5)	1000	0/3
B*	Creek	290	1/3 (38.4)	300	0/3	300	0/3	750	0/3
TC	Creek	500	0/3	480	0/3	480	0/3	750	0/3
GC	Creek	200	0/3	150	0/3	250	1/3 (38.3)	1000	0/3
M	Ephemeral pool	65	1/3 (38.4)	60	0/3	40	0/3	1000	0/3
W	Creek	90	3/3 (37.0)	60	0/3	120	0/3	1000	0/3
Pos. control*		60	3/3 (29.9)	80	3/3 (30.4)	80	3/3 (29.9)	750	0/3

*Note:* Each sample consisted of three subsamples, and each subsample was assayed in triplicate qPCR reactions. Where amplification occurred, mean Cq is provided in parentheses. * indicates sites where air samplers were also deployed. See Supporting Information S1 for site descriptions.

## 4 Discussion

We have designed an assay suitable for detecting the southern cassowary ( *Casuarius casuarius* ) across its Australian range. This assay is highly specific to the target species within Australia and highly sensitive, capable of detecting extremely low concentrations of DNA and has been successfully validated to Level 4 (substantial) on the Thalinger et al. (2021) scale. The primer and probe regions of this assay show no sequence variation between all available southern cassowary samples from Australia and Papua New Guinea, suggesting this assay is likely applicable across the species' broader Indo‐Pacific range. Outside Australia, however, further testing is required across a wider geographic area to confirm that intraspecific variation does not introduce mismatches that could reduce assay performance. The primer and probe regions of this assay also show no sequence variation with available dwarf (*n* = 5) or northern cassowary (*n* = 8) sequence, indicating that all cassowary species can be detected using this assay. Importantly, the 116 bp target region contains internal sequence variation among cassowary species, enabling Sanger sequencing to provide species‐level identification of positive eDNA detections where species co‐occur outside Australia. We demonstrate the suitability of this assay to detect southern cassowary DNA from air and water samples in both captive and natural settings in far north Queensland. Our results highlight the applicability of eDNA surveys for detecting this cryptic species across the complex habitats it occupies in Australia and demonstrate the potential utility of airborne eDNA as a monitoring tool for large terrestrial vertebrates.

Air and water eDNA sampling both successfully detected the target species in captive settings where enclosures were semi‐natural but smaller than southern cassowary home range size (Campbell et al. 2014). In captive settings, eDNA detection rates may have been influenced by the close proximity of birds to the air samplers, with the confined space of the enclosure potentially increasing the probability of airborne DNA capture compared to natural environments. This may explain the higher proportion of positive air samples detected in captive settings relative to field settings (0.33 in captivity compared with 0.11 in the field, see Table S6; Table 2). Water eDNA similarly yielded a higher proportion of positive samples in captive enclosures than in the field (1.00 in captivity compared with 0.33 in the field, Table S7; Table 3), likely due to the presence of a single, static waterbody and more frequent interaction of birds with this water source compared to their movement through larger natural landscapes. Nevertheless, positive detections at two out of three air sampling sites and at five out of six water sampling sites demonstrate that eDNA is a promising tool for detecting southern cassowaries in natural environments, supporting the utility of eDNA as a monitoring tool for this species.

Despite their large size and distinctive features, southern cassowaries are challenging to detect in the wild (McLean et al. 2017). Previous surveys and research have relied heavily on observation of scats, tracks or other signs (Westcott 1999; Westcott et al. 2014), engaging citizen scientists (Dwyer et al. 2016), or using trail cameras either with or without lures (McLean et al. 2017; Anderson et al. 2025). While these methods have shown success at detecting presence, each has limitations and the rate of missed detections is unknown. Failure to detect southern cassowary DNA at every water and air site in our field survey may reflect the broader limitations of eDNA detection (Furlan et al. 2016). For example, in field conditions, eDNA degradation and dispersion impact detection from water samples (Furlan et al. 2016; Ding et al. 2023). Although eDNA can persist in freshwater environments for up to 30 days (Ficetola et al. 2008; Goldberg et al. 2011), it can be degraded by UV exposure and temperature (Dejean et al. 2011; Barnes et al. 2014). In addition, abiotic factors such as tides, salinity, flow and dissolved and suspended solids can reduce the likelihood of detection when target organisms are present (Huang et al. 2022; Lamb et al. 2022; Mayne et al. 2024; Stevens et al. 2024; Tomke and Price 2025). While the drivers of eDNA degradation in aquatic systems are increasingly well characterised, comparable processes influencing the persistence, transport and degradation of airborne eDNA remain poorly understood, particularly in tropical environments (Johnson and Barnes 2024). Our field site is located in tropical far north Queensland, which experiences warm and humid conditions, and this is likely to have accelerated the degradation of southern cassowary DNA in our air and water samples (Tulloch et al. 2026). Further, eDNA detectability varies with season (Turner et al. 2014; Furlan et al. 2016; Minamoto et al. 2017). Our sampling coincided with heavy rainfall events towards the end of the wet season, which usually runs from November to April in far north Queensland. Sampling for eDNA during the dry season (May to October) and exploring and quantifying the impacts of seasonality and abiotic factors on southern cassowary eDNA detection will further validate and refine this technique for southern cassowary monitoring.

Improvements to the likelihood of detection of southern cassowaries with eDNA may come from increased air and water sample volume. However, sampling effort is generally constrained by filter characteristics (e.g., pore size and surface area), sampling mechanisms (e.g., hand‐pump, battery operated, or passive systems), and, for air sampling in particular, battery capacity and deployment duration (Johnson and Barnes 2024). Further exploration is warranted for air sampling to determine whether longer battery life or the use of solar panels can improve detection probabilities. Airborne eDNA is also thought to provide a relatively local signal (Tournayre et al. 2025), limiting the spatial area represented by a single sampler and likely necessitating deployment in close proximity to the target species. For southern cassowaries, fruit‐analogous lures have shown to significantly reduce required survey effort when using camera traps (McLean et al. 2017). Adopting a similar approach to lure animals into air and water sampling sites may improve eDNA detection rates, particularly in areas of unknown or suspected low density, noting, however that lures can result in non‐random movement of individuals through a landscape (Anderson et al. 2025). Given the ease of collecting eDNA samples, especially in remote locations (Young et al. 2025), this approach is well‐suited as a first‐pass detection tool to identify southern cassowary presence, which could be used to prioritise areas for targeted management actions or more intensive follow‐up surveys.

Our findings contribute to a growing body of evidence that airborne DNA can be used to detect terrestrial vertebrates (Lynggaard et al. 2022; Johnson et al. 2023; Sanchez et al. 2025) and extend this application to a large‐bodied, mobile bird species. Unlike many previous airborne DNA studies that have focused on small, enclosed, or highly controlled environments (Lynggaard et al. 2022; Garrett et al. 2023), our results demonstrate that airborne DNA can be detected in semi‐natural and open field settings. This suggests that air eDNA may be a valuable complementary tool for monitoring other elusive terrestrial vertebrates, particularly in habitats where traditional surveys are logistically challenging or invasive.

Although we have demonstrated the potential for air and water eDNA samples to detect southern cassowaries in targeted surveys at sites of known presence, the sensitivity and variability of air and water eDNA sampling for southern cassowaries in more remote habitats remain to be quantified. Understanding the probability of detection of southern cassowary eDNA in sparsely populated areas where they are likely to be at low density, particularly near the limits of the species range, is a crucial next step (Furlan et al. 2019). Species monitoring with eDNA is non‐invasive (Zhang et al. 2023) and non‐destructive (Banerjee et al. 2022) and allows for monitoring in remote and logistically challenging environments. For a keystone frugivore such as the southern cassowary, improved detection and monitoring capacity has direct implications for understanding and managing rainforest regeneration processes and ecosystem function (Mahoney et al. 2018; Albert et al. 2020). Quantifying eDNA detection probabilities for air and water samples across varying population densities and habitat types will be essential for integrating eDNA into routine monitoring and conservation programmes for southern cassowaries and other terrestrial vertebrates.

## Funding

This work was supported by the State of Queensland through the Department of the Environment, Tourism, Science and Innovation.

## Ethics Statement

All components of the works were completed with appropriate animal ethics authority (DPI ethic approval SA 2022/02/819).

## Conflicts of Interest

The authors declare no conflicts of interest.

## Author Contributions

**Peta Hill:** conceptualization (equal), data curation (lead), formal analysis (equal), methodology (equal), project administration (supporting), writing – original draft (lead), writing – review and editing (equal). **Simon Booth:** conceptualization (equal), methodology (equal), project administration (equal), writing – original draft (equal), writing – review and editing (equal). **Daniel Guymer:** project administration (equal), writing – original draft (equal), writing – review and editing (equal). **Ariella Matthews:** data curation (equal), project administration (equal), writing – review and editing (equal). **Dinouk Perera:** methodology (supporting), project administration (equal), writing – review and editing (equal). **Matt Brien:** conceptualization (equal), methodology (equal), writing – review and editing (equal). **Elise Furlan:** conceptualization (equal), formal analysis (equal), methodology (equal), project administration (equal), supervision (lead), writing – original draft (equal), writing – review and editing (equal).

## Supporting information


**Figure S1:** Location of 56 southern cassowary samples from Queensland, Australia used to design and test the C.casCytB TaqMan assay.
**Figure S2:** Approximate location of air samplers (blue dots) with respect to the resident southern cassowary at the time the samplers were deployed in an enclosure at Garners Beach Cassowary Rehabilitation Facility.
**Figure S3:** Layout of camera, air and water sampling sites for southern cassowary in Queensland, Australia.
**Table S1:** Accession numbers for cytochrome b sequences downloaded from NCBI Genbank for *Casuarius casuarius* (southern cassowary) and *Dromaius novaehollandiae* (emu) and used to design the Ccas_cytB TaqMan assay.
**Table S2:** Field trial sampling details for air and water sampling for southern cassowary in and around Mission Beach, Queensland, Australia.
**Table S3:** Base‐pair mismatches (forward primer, probe, reverse primer) and eDNAssay‐predicted assignment probabilities (AP) of cross‐amplification for the target species ( *Casuarius casuarius* ) and co‐occurring non‐target taxa within Australia and Indonesia/Papua New Guinea.
**Table S4:** Raw Cq values for 10 replicates of a serial dilution of synthetic oligonucleotide designed to match southern cassowary Cytochrome b sequence and used to determine the sensitivity of the TaqMan C.cas_CytB as per Klymus et al. (2020).
**Table S5:** Effective limits of detection with increasing analytical replicates of the southern cassowary mitochondrial Cytochrome b TaqMan assay.
**Table S6:** Mean Cq values obtained from qPCR of eDNA extracted from air samples sampled from a southern cassowary enclosure at Garners Beach Rehabilitation Facility.
**Table S7:** Mean Cq values obtained from qPCR of eDNA extracted from water sampled from a southern cassowary enclosure at Hartley's Crocodile Adventures.

## Data Availability

All data will be made available in the Supporting Information S1 upon publication. All material contained in the manuscript is original, unpublished work.
