# Supplementary material for: Air and Water Environmental DNA Enable Detection of a Large Terrestrial Bird: A Case Study of the Southern Cassowary (Casuarius casuarius)
Source: Ecol Evol. 2026 Oct 23;16(10):e74578. doi: 10.1002/ece3.74578 (PMC13693711; doi:10.1002/ece3.74578)
Supplement: Supplementary file 1 [file ECE3-16-e74578-s001.docx]

*Methods*

Table S1. Accession numbers for cytochrome b sequences downloaded from NCBI Genbank for *Casuarius casuarius* (southern cassowary) and *Dromaius novaehollandiae* (emu) and used to design the Ccas_cytB TaqMan assay. Accession numbers for *C. unappendiculatus* (northern cassowary) and *C. bennetti* (dwarf cassowary) used in the *in-silico* comparison are included. *C. casuarius* subspecies names are provided where they were included in the Genbank record although current taxonomy recognises a single species throughout the *C. casuarius* distribution (Dickinson and Remsen 2018; del Hoyo and Collar 2019; Clements et al. 2025).

| NCBI accession number | Species | Location |
| --- | --- | --- |
| NC002784.1 | *D. novaehollandiae* | Unknown |
| MN356172.1 | *D. novaehollandiae* | Unknown |
| AF338711.1 | *D. novaehollandiae* | Unknown |
| AF338713.2 | *C. casuarius* | Unknown |
| NC002778.2 | *C. casuarius* | Unknown |
| KT935327.1 | *C. casuarius* | Papua New Guinea |
| KT935325.1 | *C. casuarius sclaterii* | Papua New Guinea |
| KT935339.1 | *C. casuarius* | Papua New Guinea |
| KT935345.1 | *C. casuarius casuarius* | Papua New Guinea |
| KT935332.1 | *C. casuarius* | Papua New Guinea |
| KT935326.1 | *C. casuarius sclaterii* | Papua New Guinea |
| MN356153.1 | *C. casuarius* | Unknown |
| KT935329.1 | *C. casuarius* | Papua New Guinea |
| KT935328.1 | *C. casuarius beccarii* | Papua New Guinea |
| KT935344.1 | *C. casuarius sclaterii* | Papua New Guinea |
| KT935340.1 | *C. casuarius sclaterii* | Papua New Guinea |
| KT935336.1 | *C. casuarius sclaterii* | Papua New Guinea |
| KT935343.1 | *C. casuarius violicollis* | Papua New Guinea |
| KT935333.1 | *C. casuarius* | Papua New Guinea |
| KT935334.1 | *C. casuarius* | Papua New Guinea |
| Z49103.1 | *C. casuarius* | Unknown |
| KT935346.1 | *C. unappendiculatus* | Indonesia |
| KT935331.1 | *C. unappendiculatus* | Papua New Guinea |
| KT935330.1 | *C. unappendiculatus* | Indonesia |
| KT935338.1 | *C. unappendiculatus* | Papua New Guinea |
| KT935335.1 | *C. unappendiculatus* | Papua New Guinea |
| KT935337.1 | *C. unappendiculatus* | Papua New Guinea |
| KT935342.1 | *C. unappendiculatus* | Papua New Guinea |
| KT935341.1 | *C. unappendiculatus* | Papua New Guinea |
| U76051.1 | *C. bennetti* | Unknown |
| KT935349.1 | *C. bennetti* | Papua New Guinea |
| KT935348.1 | *C. bennetti* | Papua New Guinea |
| KT935347.1 | *C. bennetti* | Papua New Guinea |
| KT935324.1 | *C. bennetti* | Papua New Guinea |

Figure S1. Location of 56 southern cassowary samples from Queensland, Australia used to design and test the C.casCytB TaqMan assay.

*eDNA validation*

Air samples were taken from the Garners Beach Cassowary Rehabilitation Facility in Garners Beach, Queensland, Australia. Cassowaries are contained in a semi-natural environment within fenced enclosures. Air samples were taken using three air samplers custom designed and built at the University of Canberra (details in main manuscript) that were cable-tied to star pickets approximately 2m off the ground in an enclosure housing a single sub-adult female southern cassowary (Figure S2). Air sampler units were covered partially with a small 5L bucket glued to a star picket cap for protection against the rain. During this trial, two units were run for 1 hour and a third unit was run for 90 minutes. Testing of these samples using the C.cas_Cytb assay was conducted using triplicate PCRs on neat eDNA.

Figure S2. Approximate location of air samplers (blue dots) with respect to the resident southern cassowary at the time the samplers were deployed in an enclosure at Garners Beach Cassowary Rehabilitation Facility.

Water samples were taken from an enclosure housing one southern cassowary at Hartley’s Crocodile Adventures in Cairns, Queensland Australia, a wildlife sanctuary adjoining the world heritage listed wet tropics within the southern cassowary range. Cassowaries are contained in a semi-natural environment within fenced enclosures. Five water samples were taken from a pond in the enclosure using eDNA water sampling kits (see details in main manuscript). One equipment control was sampled, consisting of 750mL of tap water from the food preparation tank. The water in the food preparation area is sourced upstream of Hartley’s. Testing of these samples using the C.cas_Cytb assay was conducted using triplicate PCRs on neat eDNA.

*Mission Beach Cassowary Field Trials*

*Air and Water Field Site Descriptions*

Details of the number of days that cameras were deployed and the days on which air and water sampling took place for sites around Mission Beach Queensland where southern cassowaries are regularly sighted are included in Table S2. Sites are described below (Figure S3).

Water samples were collected by approaching one side of the waterway and collecting approximately 5 L of water using a sterile bucket. Three subsamples were then collected from the bulk water sample and filtered using custom syringe kits, as described in the main document (Section 3.2, Water eDNA sampling).

Table S2. Field trial sampling details for air and water sampling for southern cassowary in and around Mission Beach, Queensland, Australia. The number of days the cameras were deployed (grey shading), air (A) and water (W) sampling days are indicated. Site descriptions are below. Negative water sample controls were collected alongside each water sample.

|  | Air, Water and Camera Sampling | | | | | | | | | |
| --- | --- | --- | --- | --- | --- | --- | --- | --- | --- | --- |
| Day / Site | 1 | 2 | 3 | 4 | 5 | 6 | 7 | 8 | 9 | 10 |
| Site T |  |  |  |  |  |  |  | A | A | A |
|  |  |  |  |  |  |  |  | W |  |  |
| Site A |  |  |  |  |  |  |  | A |  |  |
|  |  |  |  |  |  |  |  |  |  |  |
| Site B |  |  |  |  |  |  |  |  |  | A |
|  |  |  |  |  |  |  |  |  |  | W |
| Garners Beach Cassowary Rehabilitation Facility  (Air and water positive control site) |  |  |  |  |  |  |  | A | A | A |
|  |  |  |  |  |  |  |  |  | W |  |
| Cairns DETSI base  (Air negative control site) |  |  |  |  |  |  |  | A | A | A |
|  |  |  |  |  |  |  |  |  |  |  |
| Other Water Samples | | | | | | | | | | |
| Site TC |  |  |  |  |  |  |  |  |  |  |
|  |  |  |  |  |  |  |  |  |  | W |
| Site GC |  |  |  |  |  |  |  |  |  |  |
|  |  |  |  |  |  |  |  |  | W |  |
| Site M |  |  |  |  |  |  |  |  |  |  |
|  |  |  |  |  |  |  |  |  | W |  |
| Site W |  |  |  |  |  |  |  |  |  |  |
|  |  |  |  |  |  |  |  |  | W |  |

*Site T*

Site T (Figure S3) is located at South Mission Beach in Queensland. The site consists of a patch of lawn surrounded by rainforest on private property. Cassowaries regularly walk the edge of the lawn to access a permanent water source on the property. We placed four air samplers along the path typically taken by the cassowaries. Two air samplers were located 1.4m and 1.7m from the water source with another two air samplers located 20m from these (Figure S3). We sampled air at this site for three days. We also sampled the permanent water source at this property on day 1 of air sampling.

Figure S3. Layout of camera, air and water sampling sites for southern cassowary in Queensland, Australia. Air samplers are represented by black numbered circles and distances are indicated. Blue box represents the waterbody at each site. (A) Site T in South Mission Beach with permanent waterbody; (B) Site A in Bingil Bay with transient waterbody; (C) Site B in Bingil Bay with flowing waterbody.

*Site A*

Site A (Figure S3) is located at Bingil Bay in Queensland. The site consists of rainforest surrounding a private residence. Cassowaries are regularly seen walking through the rainforest to access a transient water source on the property. We placed four air samplers along the path taken by the cassowaries, roughly equidistant all within five meters of the water source (Figure S3). We sampled air at this site for one day. The waterbody was not sampled.

*Site B*

Site B (Figure S3) is located at Bingil Bay in Queensland. The site consists of mixed paddock and grass bordered by rainforest and private residences. Cassowaries are regularly seen walking along several paths amongst the grass on the property. We placed three air samplers at the intersections of these paths (Figure S3). We sample air at this site for one day and sampled water from a flowing creek nearby on the same day.

*Water only Field Site Descriptions*

Site TC (-17.938897 / 146.057306)

This site is a small freshwater creek in the vicinity of site T. This small creek runs under an adjacent road through a concrete pipe. The sample was collected where the creek water exits the pipe into the creek bed. Several cassowaries were sighted crossing the road near this creek; an adult / offspring pair and a single adult were sighted during sampling of this site.

Site GC (-17.818076 / 146.099202)

This site is a small freshwater creek that runs behind the Garners Beach Cassowary Rehabilitation Facility. Birds are regularly sighted around the facility. An adult bird was sighted during the sampling of this site.

Site M (-17.59688 / 146.118018)

This site accumulates rainwater under a fruit tree where cassowaries are regularly seen feeding. On the day of sampling, there was considerable rainfall and the area under the tree was flooded. A single southern cassowary was sighted within approximately 100m of this tree during the sampling of this site.

Site W (-17.924178 / 146.096404)

This site is a small, estuarine creek that runs behind the local refuse centre. Cassowaries are regularly sighted in the area although no birds were sighted at the time of sampling.

*eDNA extraction – water*

DNA extraction from the water filter was performed in trace DNA facilities at the National eDNA Reference Centre following the Qiagen DNeasy blood and tissue kit with some modifications*.* To remove the preservative from the filter, the cap was removed and the preservative syringe unscrewed. The plunger on the syringe was drawn back, the syringe re-attached to the filter and the plunger depressed. This was repeated three times with the preservative discarded. The filters were allowed to air dry for 30 minutes. Caps were then replaced, and both the caps and the syringe body were sealed with parafilm where they join onto the filter. Qiagen buffer ATL (180µL) and proteinase K (20 µL) were pipetted into the syringe body and the plunger replaced and depressed 10 times to thoroughly wet the filter paper inside the filter housing. The capped filters with syringes and plungers fitted were placed in a hybridisation oven (Ratek Instruments Model No. HO35) set to 56 °C on a rocking platform set to speed 40 for 2 hours.

After digestion, the samples were removed from the oven and the ATL / Proteinase K mix was aspirated off the filter into a 10mL conical tube and the filters and syringes discarded. Subsequent steps were conducted following the manufacturers recommended protocol (<https://www.qiagen.com/au>).

*eDNA extraction - air*

eDNA air samples were extracted following the Qiagen DNeasy blood and tissue kit with some modifications. Each filter with ethanol preservative were transferred to a 50mL conical tube with sterile forceps. These were then placed in a multi-tube vortex mixer (Ratek Instruments Model No. MTV1) and vortexed for 1 hour on speed 9. The filters were then removed from the tube with sterile forceps, with as much residual liquid squeezed from the filter as possible. Samples were then pelletised by centrifuging for 12 minutes at 7,745 g. The ethanol was aspirated off and the pellet allowed to air dry for 10 minutes. 180 μL of Qiagen buffer ATL and 20 μL proteinase K were used to resuspend the pellet. Tubes were incubated overnight in a hybridisation oven (Ratek Instruments Model No. HO35) set to 56 °C on a rocking platform at speed 6. Subsequent steps were conducted following the manufacturers recommended protocol (<https://www.qiagen.com/au>).

*Results*

*Assay specificity and sensitivity*

Table S3. Base-pair mismatches (forward primer, probe, reverse primer) and eDNAssay-predicted assignment probabilities (AP) of cross-amplification for the target species (Casuarius casuarius) and co-occurring non-target taxa within Australia and Indonesia/Papua New Guinea. Taxa are ordered by decreasing AP; higher AP values indicate greater predicted likelihood of cross-amplification with the C. casuarius cytochrome b qPCR assay.

| **Location** | **Species / Taxon** | **Total mismatches (Fwd, Probe, Rev)** | **AP** |
| --- | --- | --- | --- |
| Australia | **Target: *Casuarius casuarius*** | 0 (0, 0, 0) | 0.861 |
|  | *Pezoporus occidentalis** (night parrot) | 6 (2, 2, 2) | 0.265 |
|  | *Cyclopsitta diophthalma** (double-eyed fig-parrot) | 6 (2, 2, 2) | 0.257 |
|  | *Dromaius novaehollandiae** (emu) | 7 (2, 4, 1) | 0.231 |
| Indonesia/ Papua New Guinea | **Target: *Casuarius casuarius*** (incl. subspecies *beccarii, sclaterii, violicollis*) | 0 (0, 0, 0) | 0.861 |
|  | *Chalcopsitta scintillata* (dusky lorikeet) | 4 (1, 2, 1) | 0.546 |
|  | *Tanygnathus sumatranus* (Mindanao lorikeet) | 5 (1, 2, 2) | 0.424 |
|  | *Chalcopsitta duivenbodei* (brown lory) | 6 (2, 2, 2) | 0.316 |
|  | *Lorius lory* (black-capped lory) | 6 (2, 2, 2) | 0.307 |
|  | *Psitteuteles goldiei* (Goldie's lorikeet) | 6 (2, 2, 2) | 0.307 |
|  | *Symposiachrus guttula* (New Guinea monarch) | 6 (1, 3, 2) | 0.251 |
|  | *Aegotheles tatei* (starry owlet-nightjar) | 7 (2, 3, 2) | 0.182 |
|  | *Attila citriniventris* (citron-bellied attila) | 7 (2, 3, 2) | 0.147 |

Table S4. Raw Cq values for ten replicates of a serial dilution of synthetic oligonucleotide designed to match southern cassowary Cytochrome b sequence and used to determine the sensitivity of the TaqMan C.cas_CytB as per Klymus et al (2020). NA denotes no positive amplification.

| Synthetic oligo  copies per µL | qPCR replicate | | | | | | | | | | Mean  Cq |
| --- | --- | --- | --- | --- | --- | --- | --- | --- | --- | --- | --- |
|  | 1 | 2 | 3 | 4 | 5 | 6 | 7 | 8 | 9 | 10 |  |
| 10^6^ | 19.0 | 19.2 | 18.9 | 19.1 | 19.2 | 19.0 | 19.0 | 19.0 | 19.2 | 19.3 | 19.1 |
| 10^5^ | 23.0 | 23.0 | 22.9 | 22.8 | 22.9 | 22.7 | 22.9 | 23.2 | 23.5 | 23.3 | 23.0 |
| 10^4^ | 26.8 | 26.6 | 26.7 | 27.1 | 27.0 | 26.6 | 27.1 | 26.8 | 27.3 | 27.4 | 26.9 |
| 10^3^ | 30.0 | 30.3 | 30.0 | 30.4 | 30.2 | 30.2 | 30.8 | 30.6 | 30.5 | 30.5 | 30.3 |
| 10^2^ | 32.2 | 33.7 | 33.7 | 33.9 | 33.9 | 33.8 | 34.2 | 33.9 | 33.8 | 33.7 | 33.7 |
| 10^1^ | NA | 38.5 | 39.0 | 38.3 | 36.8 | 37.0 | 37.3 | 37.1 | 37.5 | 36.8 | 37.6 |
| 10^0^ | NA | NA | 39.1 | NA | NA | NA | NA | 39.0 | NA | 39.4 | 39.1 |
| 10^-1^ | NA | NA | NA | NA | NA | NA | NA | NA | NA | NA | NA |

Table S5. Effective limits of detection with increasing analytical replicates of the southern cassowary mitochondrial Cytochrome b TaqMan assay. Values were calculated using the R code from Klymus et al. (2020) (<https://github.com/cmerkes/qPCR_LOD_Calc>).

| Number of PCR replicates | Limit of detection (copies per reaction) |
| --- | --- |
| 1 | 7.4 |
| 2 | 4.1 |
| 3 | 3.2 |
| 4 | 2.8 |
| 5 | 2.5 |
| 8 | 2.0 |

*eDNA validation*

Table S6. Mean Cq values obtained from qPCR of eDNA extracted from air samples sampled from a southern cassowary enclosure at Garners Beach Rehabilitation Facility.

| Filter number | Sample run time (hr) | Amplification success | Mean Cq |
| --- | --- | --- | --- |
| 1 | 1.5 | 0/3 | NA |
| 2 | 1 | 2/3 | 39.1 |
| 3 | 1 | 0/3 | NA |
| Control | 1 | 0/3 | NA |

Table S7. Mean Cq values obtained from qPCR of eDNA extracted from water sampled from a southern cassowary enclosure at Hartley’s Crocodile Adventures.

| Filter number | Sample volume (mL) | Amplification success | Mean Cq |
| --- | --- | --- | --- |
| 1 | 400 | 3/3 | 30.6 |
| 2 | 400 | 3/3 | 30.8 |
| 3 | 400 | 3/3 | 31.4 |
| 4 | 400 | 3/3 | 30.7 |
| 5 | 400 | 3/3 | 30.1 |
| Control | 750 | 0/3 | NA |

References

Clements JF et al. 2025. The eBird/Clements checklist of Birds of the World. https://www.birds.cornell.edu/clementschecklist/download/

Dickinson EC, Remsen JV. 2018. The Howard and Moore Complete Checklist of Birds of the World. Version 4.1. Aves Press.

del Hoyo J, Collar NJ. 2019. Handbook of the Birds of the World. Vol Version 4 Lynx Edicions,. https://doi.org/10.2307/1369764

Klymus KE et al. 2020. Reporting the limits of detection and quantification for environmental DNA assays. Environ DNA. 2(3):271–282. https://doi.org/10.1002/edn3.29
